# Plasma nitriding induced growth of Pt-nanowire arrays as high performance electrocatalysts for fuel cells

**DOI:** 10.1038/srep06439

**Published:** 2014-09-22

**Authors:** Shangfeng Du, Kaijie Lin, Sairam K. Malladi, Yaxiang Lu, Shuhui Sun, Qiang Xu, Robert Steinberger-Wilckens, Hanshan Dong

**Affiliations:** 1 School of Chemical Engineering, University of Birmingham, Edgbaston, Birmingham B15 2TT, UK; 2 School of Metallurgy and Materials, University of Birmingham, Edgbaston, Birmingham B15 2TT, UK; 3 Kavli Institute of Nanoscience, Delft University of Technology, Lorentzweg 1, 2628 CJ, Delft, the Netherlands; 4 Institut National de la Recherche Scientifique -Énergie, Matériaux et Télécommunications, Varennes QC J3X 1S2, CANADA

## Abstract

In this work, we demonstrate an innovative approach, combing a novel active screen plasma (ASP) technique with green chemical synthesis, for a direct fabrication of uniform Pt nanowire arrays on large-area supports. The ASP treatment enables in-situ N-doping and surface modification to the support surface, significantly promoting the uniform growth of tiny Pt nuclei which directs the growth of ultrathin single-crystal Pt nanowire (2.5–3 nm in diameter) arrays, forming a three-dimensional (3D) nano-architecture. Pt nanowire arrays in-situ grown on the large-area gas diffusion layer (GDL) (5 cm^2^) can be directly used as the catalyst electrode in fuel cells. The unique design brings in an extremely thin electrocatalyst layer, facilitating the charge transfer and mass transfer properties, leading to over two times higher power density than the conventional Pt nanoparticle catalyst electrode in real fuel cell environment. Due to the similar challenges faced with other nanostructures and the high availability of ASP for other material surfaces, this work will provide valuable insights and guidance towards the development of other new nano-architectures for various practical applications.

Pt-based nanostructures have been extensively investigated owing to their outstanding activity for various catalysis applications, especially for oxygen reduction reaction (ORR) and methanol oxidation reaction (MOR) in polymer electrolyte fuel cells (PEFCs)^1^,^2^,^3^. For current low-temperature fuel cells, the sluggish cathodic ORR results in the using of a high loading of expensive Pt electrocatalysts to mitigate the negative effects. The conventional state-of-the-art electrocatalysts are carbon supported Pt nanoparticles (diameters of 2–5 nm). To reduce the cost, in the past decade, many alternative non-precious metal or even metal-free electrocatalysts have been reported, e.g. N-doped carbon based and iron-based electrocatalysts, but very few of them are on a competitive level with Pt^4^,^5^,^6^,^7^,^8^,^9^,^10^. Besides the high cost, the poor durability due to Pt dissolution/agglomeration and carbon support corrosion in nanoparticle electrocatalysts also results in a short lifetime. To this end, designing highly efficient novel nanostructures with advanced nanotechnologies, the development of high-performance Pt electrocatalysts and optimisation of the fabrication of catalyst electrodes therefore become active areas of research^11^,^12^,^13^,^14^,^15^. Among various novel nanostructures, one-dimensional (1D) single-crystal Pt-nanowires have been demonstrated as an excellent new type of fuel cell electrocatalysts due to their excellent catalytic activity and stability induced by non-isotropy structures and special surface properties^16^,^17^,^18^,^19^,^20^. The synthesis of single-crystal Pt-nanowires has also been demonstrated by Xia et al^11^,^17^ and Sun et al^16^,^21^,^22^ with wet chemical process. However, due to the strict synthesis process required for the delicate control of Pt reduction kinetics, a large-area direct fabrication of uniform Pt nanowires on electrodes remains a grand challenge for practical applications^18^,^23^,^24^.

It has been well accepted that plasma surface modification is an innovative and economical technology to modify component surfaces before further processing; and among different plasma techniques, active screen plasma (ASP) could provide more efficient activation to an object with complex surfaces^25^,^26^,^27^. Furthermore, the ASP activation only modifies a very thin top layer, usually 5–50 nm, and the material removal rate due to the plasma polishing is also very low. Consequently, the dimensions and bulk features of the materials are kept intact^26^,^28^. Thus, the ASP could be a highly suitable technique for tailoring large-area support to achieve an ideal physical surface for Pt-nanowire synthesis.

In this work, the ASP activation was employed to achieve a direct large-scale surface modification to the fuel cell gas diffusion layers (GDLs). The nitrogen (N)-doping, and functional groups introduced on the support improved the surface physical properties and facilitated the formation of uniformly distributed tiny nuclei. Then, by tuning the growth reaction kinetics of the chemical synthetic process, we demonstrated a novel three-dimensional (3D) nano-architecture with Pt-nanowire arrays *in-situ* grown on the large-area GDL support. A much enhanced electrocatalytic activity was observed for this 3D nano-architecture in fuel cell environment, which could be attributed to the uniform distribution of ultrathin single-crystal Pt-nanowire arrays in the extremely thin electrocatalyst nano-architecture.

## Results

**Figure 1:**
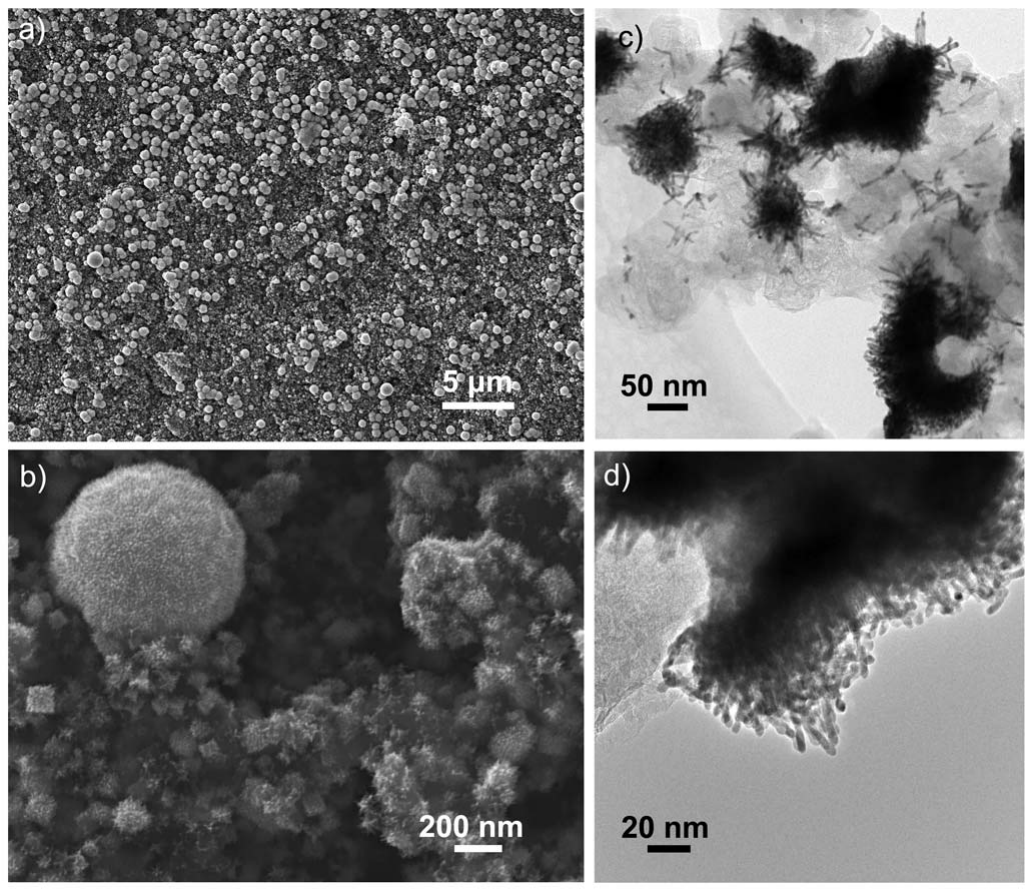
Images of Pt-nanowires grown on a pristine gas diffusion layer (GDL). (a,b) SEM images at two different magnifications. (c,d) TEM images of the Pt-nanowire superstructures formed on the pristine GDL surface.

To achieve a precise control of Pt-nanowire growth, a particular method developed by Sun et al.^16^,^21^,^22^ was chosen for this work. This method of using formic acid as reducing agent to grow Pt nanowires is very attractive due to its simplicity and easy control. The reaction was undertaken at room temperature, where a very low reduction rate of Pt ions favours the growth of {111} planes and therefore leads to the formation of Pt-nanowires. When GDL is introduced as a direct support, the rough surfaces of carbon-nanospheres contacted with solution could serve as primary sites for Pt nucleation^29^,^30^. However, as most other supports, the carbon-nanosphere surface is inert, which is usually required to be activated when used as catalyst support^2^,^31^. Consequently, on the pristine support surface, Pt nuclei could only form on the very limited sites, finally resulting in a non-uniform Pt-nanowire growth on the support surface (Figure 1). It can be seen that there are many huge aggregates formed, which have a diameter of ca. 1 μm or even larger, providing little contribution to the catalytic ability. Finally, only short Pt-nanowires were obtained with a low surface coverage on carbon nanospheres, and most of them also assembled to 3D superstructures with a size of 50–200 nm, as shown in SEM and TEM images in Figure 1b–d. The short Pt nanowires in superstructures have a diameter of ca. 4 nm in average and this is consistent with those reported in literature^21^,^22^. In this case, these non-uniform Pt-nanowires will result in a low Pt utilization and a poor catalytic performance could be expected.

**Figure 2:**
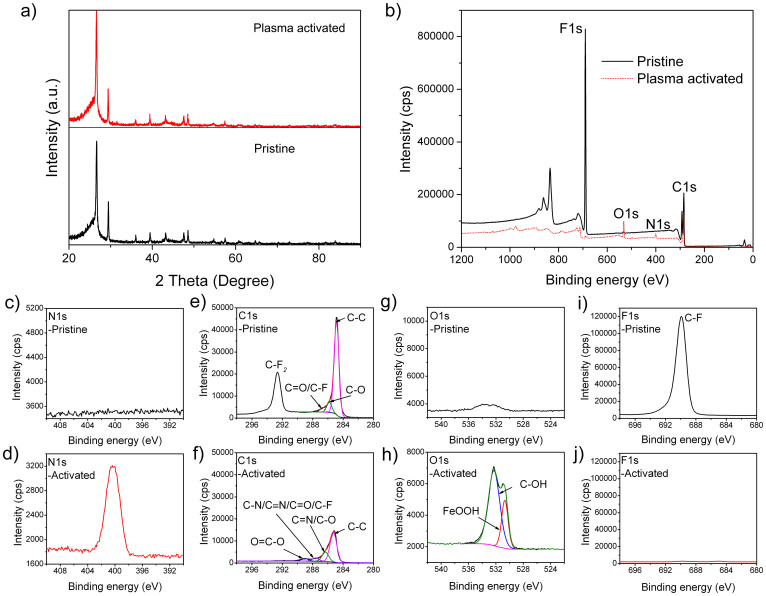
XRD and XPS patterns of GDL supports. (a) XRD patterns of GDL supports before and after the ASP activation. (b) XPS survey, (c,d) N1s, (e,f) C1s, (g,h) O1s, and (i,j) F1s spectra of the support GDL surfaces. (c,e,g,i) pristine GDL support and (d,f,h,j) the ASP activated GDL support.

In order to achieve highly active support surface, the active screen plasma (ASP) treatment was conducted to the 5 cm^2^ GDL in a gas mixture of H_2_/N_2_ at 120°C for 10 min. Figure 2a displays XRD patterns for the pristine and ASP activated GDLs. Nearly identical peaks were obtained for two GDL supports, confirming the only surface improvement feature of this technique without altering the bulk structure. To further investigate the activation role of the ASP treatment to the support surface, a comparison was conducted by XPS analysis to the sample before and after the activation (Figure 2b–j). The main contribution from this ASP activation is functional groups formed on the GDL surface, e.g. C-N, C = N, C≡N and O-H, as shown by the XPS survey, N1s, C1s and O1s spectra. After the ASP activation, an obvious N1s peak appeared at 400.3 eV for the activated support surface (Figure 2b and d), as also been suggested by the appearance of C-N/C≡N peak in C1s spectrum (Figure 2f), indicating the incorporation of nitrogen (N)-containing groups^32^,^33^, whereby the overall nitrogen content is ca. 6.2% (Table S1). This nitriding of the surface could significantly improve the Pt nucleation on the surface. It could provide more defects on the carbon nanosphere surface, thus confining the Pt atoms to form tiny nuclei and further leading to the anisotropic growth of ultrathin Pt-nanowires with a smaller diameter^34^. The ultrathin Pt-nanowires possess relative larger specific surface area, providing better activities in catalytic reactions. Furthermore, regarding the carbon-nanosphere support itself, the N-doping changes its electronic band, thus giving itself extra catalytic activity in catalysis reactions^6^,^35^. Another role of this N-doping is that the incorporation of N-containing groups leads to a high surface activity, enabling a better contact between the support surface and the reaction solution, thus facilitating the formation of a large amount of nuclei on support surface. Besides the N-containing groups, the O-H functional group was also detected at 532.3 eV in the O1s spectrum (Figure 2h), which will also contribute to the improvement of the surface activity. Another new component also appeared at 530.7 eV in the O1s spectrum, corresponding to oxygen atoms in iron-oxide contamination from the ASP activation^33^. The trace amount of iron-oxide could be easily removed in the acid environment during the following Pt-nanowire growth process and thus had very little influence on the final catalytic properties^36^; Another benefit for this special GDL support might come from the partially removing of PTFE from the surface indicated by the decline of the F1s spectra at 689.9 eV (Figure 2i–j) and the C1s spectrum for PTFE at 292.6 eV due to the CF_2_-groups in the polymer chains (Figure 2e and f)^37^. This will also enhance the surface activity in some degree.

**Figure 3:**
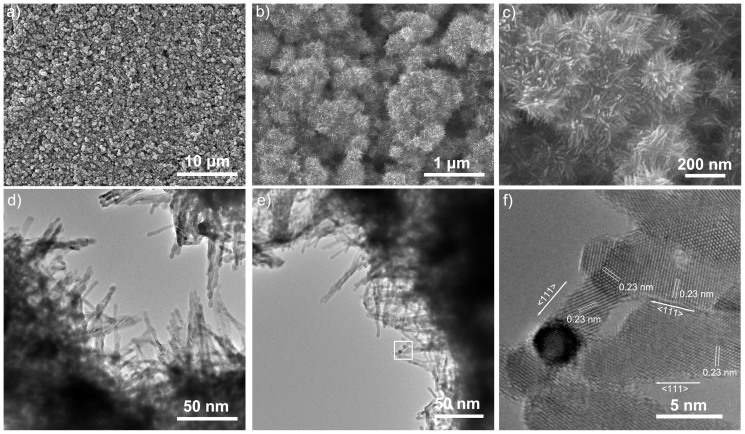
Images of Pt-nanowires grown on the ASP-treated GDLs. (a–c) SEM images of a 3D nano-architectured catalyst layer with Pt-nanowire arrays in-situ grown on the ASP activated GDL support surface, at three different magnifications. The support area is 5 cm^2^. (d–f) TEM and HR-TEM images of Pt-nanowires in the nano-architectured catalyst layer. (f) shows a HR-TEM image of the part specified by the white square in (e), indicating the single-crystal nanowires with the growth direction along the <111> axis.

The SEM images of Pt-nanowire arrays in-situ grown on a 5 cm^2^ ASP activated GDL support are shown in Figure 3a–c. It can be seen that uniform Pt-nanowire arrays were obtained through the entire GDL support surface. Pt-nanowire arrays follows the ups and downs along the carbon nanosphere surface, forming an extremely thin 3D nano-architectured catalyst layer. The obtained Pt-nanowires had a length of about 50–200 nm. Figure 3d–f further shows TEM and HR-TEM images of these Pt nanowires. All of the Pt nanowires projected randomly outwards from the support surface. They had a small diameter of 2.5–3 nm, with a lattice spacing of 0.23 nm, which agrees with that of {111} planes of the bulk Pt crystal, indicating the single-crystal feature of these Pt-nanowires growing along the <111> direction. XRD analysis (Figure S1) confirmed the face-centered-cubic (fcc) structure of these Pt nanowires^11^,^22^,^38^.

**Figure 4:**
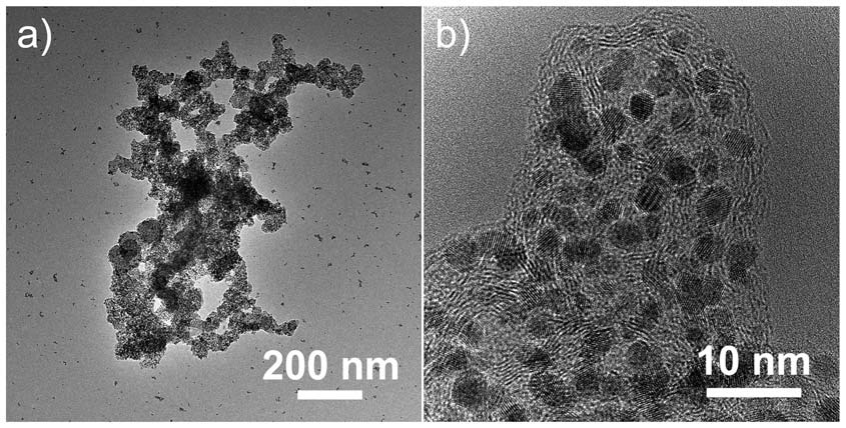
TEM images of Pt nanoparticles in the conventional catalyst electrode (Johnson-Matthey DMFC cathode).

**Figure 5:**
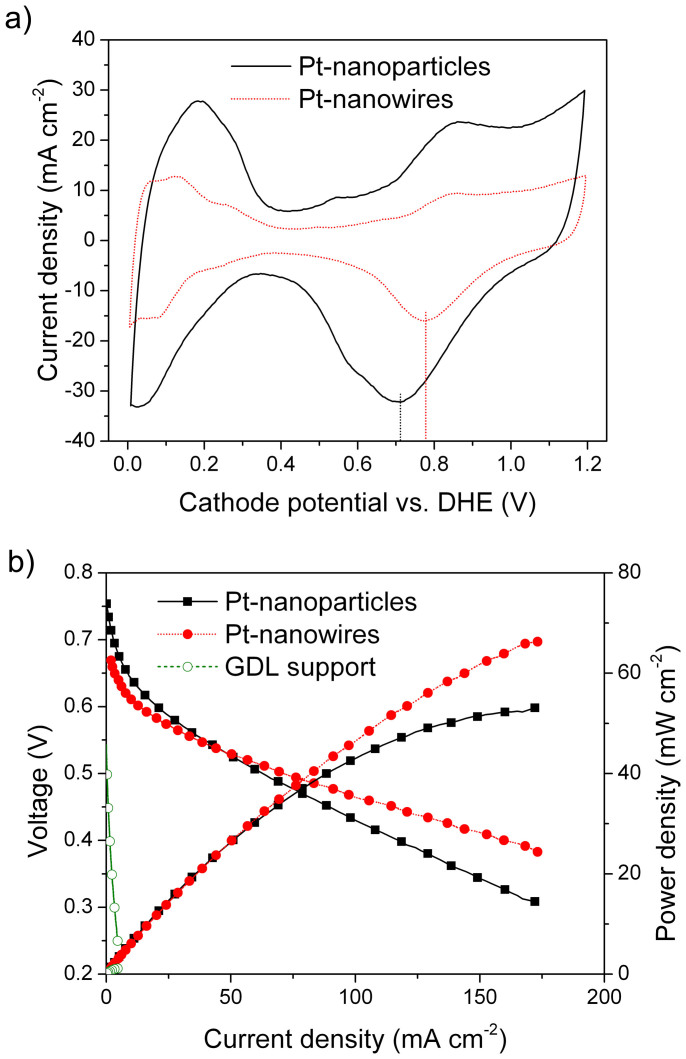
Catalytic performance of catalyst electrodes. (a) Cathode cyclic voltammetry (CV) and (b) polarization curves for the 3D nano-architectured electrode with Pt-nanowire arrays (2 mg_Pt_/cm^2^), the conventional Pt-nanoparticle catalyst electrode (Johnson-Matthey DMFC cathode, 4 mg_Pt_/cm^2^), and the empty ASP-treated GDL support tested in a 5 cm^2^ DMFC.

The practical catalytic activities of the as-prepared 3D nano-architectured catalyst electrode with Pt-nanowire arrays were tested directly as cathodes in direct methanol fuel cells (DMFCs). The reason for choosing DMFC rather than hydrogen/air PEFC is that a much higher Pt loading (e.g. 4 mg/cm^2^) is usually needed in DMFC cathode than that in PEFC cathodes (0.4 mg/cm^2^ or even less). An effective reducing of the Pt amount in DMFCs will be much prominent in lowing the catalyst cost. The as-prepared 3D Pt nanowire nano-architecture with GDL support was used directly as the cathode for the fabrication of the membrane electrode assembly (MEA). For comparison, a MEA with a conventional state-of-the-art Pt nanoparticle cathode (Johnson-Matthey, 4.0 mg_Pt_/cm^2^) was also fabricated simultaneously using the same procedure. TEM images of the Pt nanoparticles in the conventional cathode are shown in Figure 4. The Pt nanoparticles have a primary diameter of ca. 4 nm in average, with light aggregation. The electrochemical surface area (ECSA) of the nanoparticle and nanowire electrodes is compared by electrode cyclic voltammetry (CV) analysis in DMFCs (Figure 5a). The different shapes of the hydrogen under-potential adsorption and de-sorption (H-UPD) peaks observed between 0 and 0.4 V reveal the different Pt exposure crystal facets between Pt nanowires and nanoparticles. Furthermore, Pt-nanowire electrode showed a Pt oxide reduction peak at a much higher potential than Pt-nanoparticle electrode (0.78 versus 0.71 V), revealing a weakening of the bond between oxygen containing species and the Pt surface on Pt nanowires, a property linked to the increased ORR performance. After normalization by the Pt mass, very close ECSAs were obtained for both electrodes, which are 25.4 m^2^/g for the Pt-nanowire electrode and 25.8 m^2^/g for the Pt-nanoparticle one, respectively. The similar ECSA of the two electrodes agrees well with the different morphologies and sizes of the ultra-thin single-crystal Pt nanowires and the conventional Pt nanoparticles.

As we know, compared with Pt nanoparticles, single-crystal Pt-nanowires possess many morphological advantages, including i) the preferred <111> growth direction results in a preferential exposure of certain crystal facets. It has been confirmed that the different low-index surfaces have markedly different activities, e.g. the change in activity between the least active (100) and the most active (111) surfaces being greater than an order of magnitude^39^; ii) fewer surface defects borne by the nanowires which have a closer resemblance to the surface of large single Pt crystals that exhibit even higher ORR specific activities^21^. Furthermore, this smooth atomic surfaces with Pt nanowires have a smaller number of low coordination atoms as compared with those of Pt nanoparticles, thus possess a reduced interaction with OH species. This is also confirmed by the higher Pt oxide reduction peak in the CV analysis here (Figure 5a). Because the adsorbed OH_ad_ species on the Pt surface could block the active surface for O_2_ adsorption and thus have a negative impact on the ORR, these single-crystal Pt nanowires with a lower coverage of OH_ad_ species help improve the ORR kinetics, finally leading to enhanced activities^40^; and iii) the anisotropic 1D morphology of Pt nanowires can improve mass transport and catalyst utilization by facilitating the reaction kinetics and improving the O_2_ diffusion to Pt surface^41^. In this case, considering the similar ECSA but the unique properties of the single-crystal Pt-nanowires, a higher catalytic performance was as expected for the 3D Pt nanowire nano-architecture.

The polarization curves for MEAs are shown in Figure 5b. With only half the Pt metal loading, 2 mg_Pt_/cm^2^, the as-prepared 3D nano-architectured electrode exhibited a comparable power density than the Pt-nanoparticle one with a Pt loading of 4 mg_Pt_/cm^2^. At 0.4 V — the practical operating voltage of DMFCs — the power density is 64 mW/cm^2^ for the Pt-nanowire electrode, which is 36% higher than 47 mW/cm^2^ of the Pt nanoparticle electrode at a double Pt loading. At a larger current density and the lower operating voltage, this improvement became much prominent. But, at a high-voltage range above 0.53 V, the Pt-nanowire electrode showed a slightly lower current density, which could be contributed to the better kinetics of a larger Pt loading in the Pt-nanoparticle electrode^42^,^43^. To further understand the role of the ASP-treated GDL support in the Pt nanowire electrode, a MEA was also fabricated with the empty ASP-treated GDL as the cathode, but free of Pt nanowires. The polarization curve is also included in Figure 5b. It can be seen that even without Pt catalysts, the electrode still shows some ORR performance itself. A power density of 0.6 mW/cm^2^ is obtained at 0.4 V, which should be attributed to the N-doping carbon-nanosphere surface in the GDL. However, compared with Pt nanoparticle and Pt nanowire electrodes, this power value is extremely smaller.

## Discussion

Based on this 3D nano-architectures with uniform Pt-nanowire arrays in-situ grown on the large-area GDL support, we achieved a true high performance catalyst electrode in practical applications. The enhanced activities of the 3D nano-architectures compared with conventional Pt-nanoparticle electrodes could be due to several factors, including:

- The ASP activation introduces various functional groups, e.g. C-N, C = N and O-H on the support surface (Figure 2), which facilitate the formation of Pt nuclei thus enabling a uniform growth of Pt-nanowire arrays on the support surface (Figure 3). This nano-architecture effectively reduces the huge aggregates formed in the catalyst layer thus improving the catalyst utilisation;
- The ASP nitriding process introduced N-doping and more defects to the support surface. These defects confine the Pt atoms in reaction to form small nuclei, resulting in the growth of ultrathin Pt-nanowires with diameters of 2.5–3.0 nm (Figure 3). The reduced diameter increases the metal surface area, leading to an ECSA as large as that of Pt-nanoparticles in the state-of-the-art commercial electrodes.
- The electrocatalyst layer consisting of only Pt-nanowire arrays has an extremely small thickness. The extremely thin catalyst layer with ordered Pt-nanowire arrays effectively reduces the transfer path of electrons and protons going to the reaction site and out of the catalyst layer, bringing in a higher catalyst utilization; furthermore, this 3D nano-architecture significantly reduces the mass transfer resistance within the thin catalyst layer, which becomes predominant in particular at a large current density range (Figure 5b)^13^,^14^;
- As mentioned above in results, the single-crystal effect of Pt-nanowires as compared with Pt nanoparticles, such as preferred <111> growth direction, 1D morphology^41^, the preferential exposure of certain crystal facets^39^, fewer surface defects^21^ and the reduced interaction with OH species^40^, contributes to the enhanced ORR activities and CO tolerance in DMFCs.

The ASP activation technique and the post chemical growth procedure are both highly scalable and eco-friendly, together with the high performance obtained, which offer the great potential of these catalyst electrodes for practical applications. Furthermore, many other nanostructures, e.g. N-, or S-doped CNTs or graphene, anisotropic metal and metal oxide nanostructures, face similar challenges as Pt-nanowires in practical applications, in addition with the high availability of the active plasma screen treatment for most materials surface, therefore, our understanding gained here could provide valuable insight towards the design of new nano-architectures from other types nanostructures for various catalysis applications.

## Methods

### Materials

All chemicals were used as received without any further purification. Sulphuric acid (H_2_SO_4_), hydrogen peroxide (H_2_O_2_), tetrahydrofuran (THF), ethanol (C_2_H_6_O), hydrogen hexachloroplatinic acid (H_2_PtCl_6_·6H_2_O, 99.95%) and formic acid (CH_2_O_2_, 98%) were purchased from Sigma-Aldrich. SIGRACET® GDL 35BC was used as the direct GDL support for growing Pt-nanowires. Johnson-Matthey (JM) DMFC cathode (Pt nanoparticles, 4 mg_Pt_/cm^2^) and Anode (4 mg_PtRu_/cm^2^) from Alfa Aesar were taken as the commercial state-of-the-art electrodes. Nafion® 117 membrane and DE 1021 (10 wt%) ionomer solution were used as received from Ion Power. All aqueous solutions were prepared with ultra pure water (18.2 MΩ.cm) from a Millipore water system.

### Active screen plasma (ASP) activation

SIGRACET® GDL 35BC carbon paper was used as the direct support for growing Pt-nanowires. The carbon paper was cut to 5 cm^2^ pieces, the same size used in fuel cell testing. The ASP activation was conducted in an active screen plasma system converted from a direct current (DC) plasma nitriding furnace (40 kW, Klöckner Ionon), the details of which can be found elsewhere^33^. A gas mixture of 25% N_2_ and 75% H_2_ was used for the treatment. When a cathodic potential of about 500 V was applied to a stainless steel mesh screen surrounding the working table at a floating potential, plasma was formed on the screen to activate the carbon papers on the working table at 120°C for 10 min under 2 mbar (200 Pa).

### Pt-nanowire growth

Typically, to grow Pt-nanowires on a GDL surface, the pristine or ASP activated carbon paper pieces were first cleaned by sonication in ethanol aqueous solutions (5 vol%), and then immersed in an aqueous solution of H_2_PtCl_6_ and formic acid at room temperature. Normally, for the growth of 2 mg/cm^2^ Pt-nanowires, 26.5 mg H_2_PtCl_6_·6H_2_O (10 mg Pt, 2 mg/cm^2^ on a 5 cm^2^ GDL) and 0.80 ml formic acid were added to 32 mL water. The samples were stored at room temperature for 72 hours. After completion of the growth of nanowires on the substrates, the samples were rinsed with water and dried overnight at 40°C. The Pt loading of 2 mg/cm^2^ was controlled by monitoring the Pt precursor mass versus the support area. The carbon paper with in-situ grown Pt-nanowire arrays was used directly as catalyst electrode in DMFC testing below.

### Membrane electrode assembly (MEA) and single cell fabrication

Nafion® 117 membrane was pre-activated by boiling for 1 hour in 3% H_2_O_2_, water, 0.5 M H_2_SO_4_ and water, respectively. Then the as-prepared Pt-nanowire electrodes were assembled as cathodes with Nafion® 117 membranes to produce MEAs with commercial JM anodes (4 mg_PtRu_/cm^2^), onto which a thin layer of Nafion ionomer was painted using an IPA solution of Nafion® DE 1021 (volume ratio of DE 1021 to IPA = 1:2) followed by drying for 1 hour at room temperature. The electrodes were then hot-pressed against the membrane at 135°C for 2 min under a pressure of 50 kg/cm^2^. For comparison purposes, MEAs were simultaneously fabricated with Nafion® 117 membrane and commercial Pt-nanoparticle cathode (JM, 4 mg_Pt_/cm^2^) and anode, as well as a one with empty ASP-treated GDL as cathode. The hard Teflon film with a thickness of 254 μm was used as gasket material in the DMFC hardware. The MEA was sandwiched between two graphite flow field plates to form a single cell with an active electrode area of 5 cm^2^.

### Electrochemical characterization

Cathode cyclic voltammetry (CV) and single-cell test were carried out at 75°C. CV was recorded with the cathode fed with ultra-pure water at 1 mL/min, and the anode fed with non-humidified hydrogen at a flow rate of 100 sccm serving as both reference and counter electrode, also designated as a dynamic hydrogen electrode (DHE). Then the cathode potential was cycled between 0 and 1.2 V versus DHE at 20 mV/s for 5 cycles, and the fifth cycle was recorded. Single cell testing was performed by obtaining polarisation curves using an EZstat-Pro system with the maximum current of 1 A. The anode was fed with 1 mol/L methanol at a flow rate of 1 mL/min. The cathode was fed with non-humidified air with a flow rate of 100 sccm without backpressure. Then the cell voltage was looped between 0.25 V and OCV at 3 mV/s for 5 cycles, and the fifth cycle was recorded.

### Physical characterization

Scanning electron microscopy (SEM) analysis of the as-prepared nano-architectures was performed with a field emission scanning electron microscope (FE-SEM, JEOL 7000F, operating at 20 kV). High-resolution transmission electron microscopy (HR-TEM) images were recorded on a FEI Tecnai F20ST microscope operating at 200 kV. Samples for TEM were prepared by scraping the top layer off the GDE pieces, dispersing in ethanol, and placing a drop of the dispersion on a Cu grid covered with carbon film. X-Ray diffraction (XRD) patterns were obtained with a Siemens 5005 X-ray diffractometer using Cu *Kα* radiation at *λ* = 1.5418 Å. X-Ray photoelectron spectroscopy (XPS) characterization was performed on an XPS spectrometer (VG Escalab 250) by using a high intensity monochromated Al *Kα* source.

## Author Contributions

S.F.D., K.J.L. and Y.X.L. synthesized the catalysts, prepared the fuel cell test materials and/or conducted fuel cell tests and processed the data. K.J.L. and H.S.D. conducted plasma treatment and processed the data. S.F.D., K.J.L., S.K.M., Y.X.L. and Q.X. performed physical characterizations (XRD, XPS, SEM and TEM). S.F.D. and H.S.D. contributed to the planning and coordination of the project. S.F.D., H.S.D., S.H.S. and R.S.W. wrote and edited the manuscript and Supplementary Information. All authors contributed to discussions about the results and the manuscript. The project was conceived and supervised by S.F.D.

## Supplementary Material

Supplementary Information Supplementary Infomation

## References

[b1] Zhang J. PEM Fuel Cell Electrocatalysts And Catalyst Layers: Fundamentals and Applications. (Springer, London, 2008).

[b2] Zhang J. & Liu H. Electrocatalysis of direct methanol fuel cells: from fundamentals to applications. (Wiley-VCH; Chichester: John Wiley [distributor], Weinheim, 2009).

[b3] Katsounaros I., Cherevko S., Zeradjanin A. R. & Mayrhofer K. J. J. Oxygen Electrochemistry as a Cornerstone for Sustainable Energy Conversion. Angew Chem Int Edit 53, 102–121 (2014). 10.1002/anie.201306588 24339359

[b4] Li Y. G. *et al.* An oxygen reduction electrocatalyst based on carbon nanotube-graphene complexes. Nat Nanotechnol 7, 394–400 (2012). 22635099 10.1038/nnano.2012.72

[b5] Bashyam R. & Zelenay P. A class of non-precious metal composite catalysts for fuel cells. Nature 443, 63–66 (2006). 16957726 10.1038/nature05118

[b6] Gong K. P., Du F., Xia Z. H., Durstock M. & Dai L. M. Nitrogen-Doped Carbon Nanotube Arrays with High Electrocatalytic Activity for Oxygen Reduction. Science 323, 760–764 (2009). 19197058 10.1126/science.1168049

[b7] Lefevre M., Proietti E., Jaouen F. & Dodelet J. P. Iron-Based Catalysts with Improved Oxygen Reduction Activity in Polymer Electrolyte Fuel Cells. Science 324, 71–74 (2009). 19342583 10.1126/science.1170051

[b8] Wu G., More K. L., Johnston C. M. & Zelenay P. High-Performance Electrocatalysts for Oxygen Reduction Derived from Polyaniline, Iron, and Cobalt. Science 332, 443–447 (2011). 21512028 10.1126/science.1200832

[b9] Yang W., Fellinger T. P. & Antonietti M. Efficient Metal-Free Oxygen Reduction in Alkaline Medium on High-Surface-Area Mesoporous Nitrogen-Doped Carbons Made from Ionic Liquids and Nucleobases. J Am Chem Soc 133, 206–209 (2011). 21155583 10.1021/ja108039j

[b10] Proietti E. *et al.* Iron-based cathode catalyst with enhanced power density in polymer electrolyte membrane fuel cells. Nat Commun 2 (2011). 10.1038/ncomms1427 21811245

[b11] Lee E. P. *et al.* Growing Pt nanowires as a densely packed array on metal gauze. J Am Chem Soc 129, 10634–10635 (2007). 17685620 10.1021/ja074312e

[b12] Du S. F. A Facile Route for Polymer Electrolyte Membrane Fuel Cell Electrodes with in situ Grown Pt Nanowires. J Power Sources 195, 289–292 (2010).

[b13] Debe M. K. Nanostructured Thin Film Electrocatalysts for PEM Fuel Cells - A Tutorial on the Fundamental Characteristics and Practical Properties of NSTF Catalysts. Ecs Transactions 45, 47–68 (2012).

[b14] Debe M. K. Electrocatalyst approaches and challenges for automotive fuel cells. Nature 486, 43–51 (2012). 22678278 10.1038/nature11115

[b15] Holdcroft S. Fuel Cell Catalyst Layers: A Polymer Science Perspective. Chem Mater 26, 381–393 (2013).

[b16] Sun S., Yang D., Zhang G., Sacher E. & Dodelet J. P. Synthesis and characterization of platinum nanowire-carbon nanotube heterostructures. Chemistry of Materials 19, 6376–6378 (2007).

[b17] Lee E. P. *et al.* Electrocatalytic Properties of Pt Nanowires Supported on Pt and W Gauzes. Acs Nano 2, 2167–2173 (2008). 19206464 10.1021/nn800458p

[b18] Du S. Pt-based nanowires as electrocatalysts in proton exchange fuel cells. International Journal of Low-Carbon Technologies 7, 44–54 (2012).

[b19] Wang R. Y. *et al.* Controlled Growth of Platinum Nanowire Arrays on Sulfur Doped Graphene as High Performance Electrocatalyst. Sci Rep-Uk 3 (2013). 10.1038/srep02431 PMC3743054 23942256

[b20] Xia B. Y., Bin Wu H., Yan Y., Lou X. W. & Wang X. Ultrathin and Ultralong Single-Crystal Platinum Nanowire Assemblies with Highly Stable Electrocatalytic Activity. J Am Chem Soc 135, 9480–9485 (2013). 23742152 10.1021/ja402955t

[b21] Sun S. H., Jaouen F. & Dodelet J. P. Controlled Growth of Pt Nanowires on Carbon Nanospheres and Their Enhanced Performance as Electrocatalysts in PEM Fuel Cells. Adv Mater 20, 3900–3904 (2008).

[b22] Sun S. H. *et al.* A Highly Durable Platinum Nanocatalyst for Proton Exchange Membrane Fuel Cells: Multiarmed Starlike Nanowire Single Crystal. Angew Chem Int Edit 50, 422–426 (2011). 10.1002/anie.201004631 21082633

[b23] Koenigsmann C. & Wong S. S. One-dimensional noble metal electrocatalysts: a promising structural paradigm for direct methanol fuel cells. Energy & Environmental Science 4, 1161–1176 (2011).

[b24] Tiwari J. N., Tiwari R. N. & Kim K. S. Zero-dimensional, one-dimensional, two-dimensional and three-dimensional nanostructured materials for advanced electrochemical energy devices. Prog Mater Sci 57, 724–803 (2012).

[b25] Alves C. *et al.* Use of cathodic cage in plasma nitriding. Surf Coat Tech 201, 2450–2454 (2006).

[b26] Corujeira Gallo S. Active screen plasma surface engineering of austenitic stainless steel for enhanged tribological and corrosion properties. (University of Birmingham, Birmingham, 2009).

[b27] Li C. X., Bell T. & Dong H. A study of active screen plasma nitriding. Surf Eng 18, 174–181 (2002).

[b28] Arefi F., Andre V., Montazerrahmati P. & Amouroux J. Plasma Polymerization and Surface-Treatment of Polymers. Pure Appl Chem 64, 715–723 (1992).

[b29] Gouldstone A., Van Vliet K. J. & Suresh S. Nanoindentation - Simulation of defect nucleation in a crystal. Nature 411, 656–656 (2001). 11395759 10.1038/35079687

[b30] Brune H. Microscopic view of epitaxial metal growth: nucleation and aggregation. Surf Sci Rep 31, 121–229 (1998).

[b31] Antolini E. Carbon supports for low-temperature fuel cell catalysts. Appl Catal B-Environ 88, 1–24 (2009).

[b32] Kaklamani G. *et al.* Active screen plasma nitriding enhances cell attachment to polymer surfaces. Appl Surf Sci 273, 787–798 (2013).

[b33] Fu X., Jenkins M. J., Sun G. M., Bertoti I. & Dong H. S. Characterization of active screen plasma modified polyurethane surfaces. Surf Coat Tech 206, 4799–4807 (2012).

[b34] Sun S. H. *et al.* Ultrathin single crystal Pt nanowires grown on N-doped carbon nanotubes. Chemical Communications 7048–7050 (2009). 19904390 10.1039/b916080a

[b35] Pylypenko S. *et al.* Nitrogen: unraveling the secret to stable carbon-supported Pt-alloy electrocatalysts. Energy & Environmental Science 6, 2957–2964 (2013).

[b36] Sun S. H. *et al.* Template- and surfactant-free room temperature synthesis of self-assembled 3D pt nanoflowers from single-crystal nanowires. Adv Mater 20, 571–574 (2008).

[b37] Schurmann U., Hartung W., Takele H., Zaporojtchenko V. & Faupel F. Controlled syntheses of Ag-polytetrafluoroethylene nanocomposite thin films by co-sputtering from two magnetron sources. Nanotechnology 16, 1078–1082 (2005).

[b38] Chen J. Y., Herricks T., Geissler M. & Xia Y. N. Single-crystal nanowires of platinum can be synthesized by controlling the reaction rate of a polyol process. J Am Chem Soc 126, 10854–10855 (2004). 15339165 10.1021/ja0468224

[b39] Stamenkovic V. R. *et al.* Improved oxygen reduction activity on Pt3Ni(111) via increased surface site availability. Science 315, 493–497 (2007). 17218494 10.1126/science.1135941

[b40] Zhang J. L., Vukmirovic M. B., Xu Y., Mavrikakis M. & Adzic R. R. Controlling the catalytic activity of platinum-monolayer electrocatalysts for oxygen reduction with different substrates. Angew Chem Int Edit 44, 2132–2135 (2005). 10.1002/anie.200462335 15714453

[b41] Chen Z. W., Waje M., Li W. Z. & Yan Y. S. Supportless Pt and PtPd nanotubes as electrocatalysts for oxygen-reduction reactions. Angew Chem Int Edit 46, 4060–4063 (2007). 10.1002/anie.200700894 17476642

[b42] Leimin X., Shijun L., Lijun Y. & Zhenxing L. Investigation of a Novel Catalyst Coated Membrane Method to Prepare Low-Platinum-Loading Membrane Electrode Assemblies for PEMFCs. Fuel Cells 9, 101–105 (2009).

[b43] Du S. F. & Pollee B. G. Catalyst loading for Pt-nanowire thin film electrodes in PEFCs. Int J Hydrogen Energ 37, 17892–17898 (2012).

