# Supplementary material for: Plasma nitriding induced growth of Pt-nanowire arrays as high performance electrocatalysts for fuel cells
Source: Sci Rep. 2014 Sep 22;4:6439. doi: 10.1038/srep06439 (PMC4170194; doi:10.1038/srep06439)
Supplement: Supplementary Information — Supplementary Infomation [file srep06439-s1.doc]

Supplementary Info

Plasma nitriding induced growth of Pt-nanowire arrays as high performance electrocatalyst for fuel cells

Shangfeng Du*^,1^, Kaijie Lin^2^, Sairam K Malladi^3^, Yaxiang Lu^1^, Shuhui Sun^4^, Qiang Xu^3^, Robert Steinberger-Wilckens^1^ and Hanshan Dong*^,2^


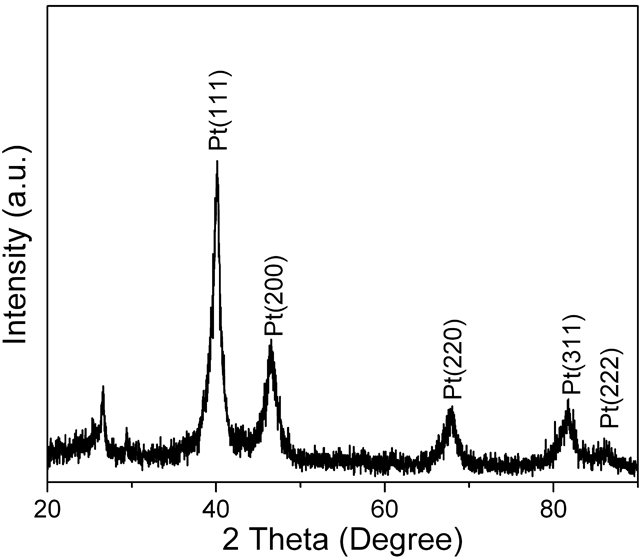


Figure S1 | XRD pattern of 3D nano-architecture with Pt-nanowire arrays on GDL support.

Table S1 | The element contents from XPS analysis for the GDL support surface before and after the active screen plasma activation.

| Element (Atomic %) | C1s | F1s | Fe1s | N1s | O1s |
| --- | --- | --- | --- | --- | --- |
| Pristine GDL | 60.97 | 38.48 | 0 | 0 | 0.52 |
| Activated GDL | 75.20 | 2.12 | 2.62 | 6.22 | 13.83 |
